# Recycling Ag SERS-substrates from strongly chemisorbing molecules

**DOI:** 10.1039/d6na00022c

**Published:** 2026-03-12

**Authors:** Marc Bröckel, Diana Heberle, Alfred J. Meixner, Kai Braun

**Affiliations:** a Institute of Physical and Theoretical Chemistry, University of Tübingen Auf der Morgenstelle 18 72076 Tübingen Germany kai.braun@uni-tuebingen.de alfred.meixner@uni-tuebingen.de; b Center for Light-Matter Interaction, Sensors & Analytics LISA+, University of Tübingen Auf der Morgenstelle 15 72076 Tübingen Germany

## Abstract

The molecular fingerprint is a viable tool for molecular detection in all kinds of research and industrial fields. Surface-enhanced Raman spectroscopy (SERS) combines this with a high sensitivity of up to single-molecules. The plasmonic active SERS substrates most commonly consist of nanostructured noble metals on which analytes adsorb. Therefore, most SERS substrates are single use, which limits the standardized commercial usability. To overcome this issue, many researchers are investigating different methods to recycle SERS substrates like solvent washing, thermal annealing or chemical reactions. However, many environmental toxins, biomolecules or drugs are expected to exhibit strong chemisorption on metal surfaces. Harsher techniques such as plasma cleaning are required. While Ar- or O_2_-plasmas have already been tested for Au substrates, plasma-recycling of Ag substrates is difficult due to fast Ag oxidation. In this work, we present a plasma-based cleaning procedure (O_2_-plasma oxidation with subsequent H_2_-plasma reduction) which completely removes the tested analytes rhodamine 6G (R6G), 2-mercaptobenzothiazole (MBT) and 1,2-benzenedithiol (BDT). We report the analyte SERS spectra for 20 subsequent recycling steps where the analytical enhancement factors (AEFs) first increase for the first ten recycling steps and then decrease again. We correlate this trend in AEFs with the nanoscale structural changes of the substrate by means of SEM images. The presented method is easily applicable to similar kinds of Ag-substrates and any type of analyte molecules. SERS substrate recycling is one step towards waste regulation and reproducible AEFs.

## Abstract 2

The molecular fingerprint is a viable tool for molecular detection in all kinds of research and industrial fields.

## Graphical abstract

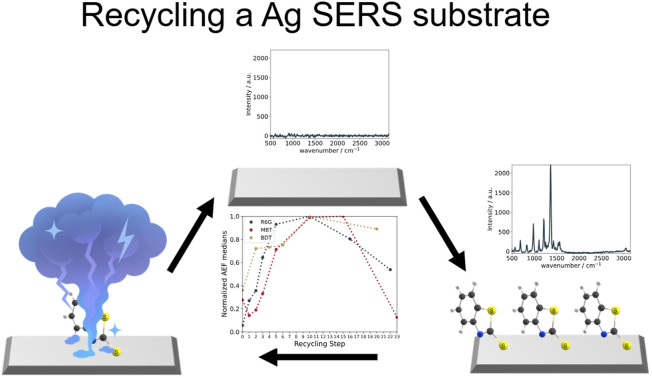

## Introduction

The ability to detect and analyze molecules with high specificity plays an important role in many scientific and industrial fields, such as healthcare,^1–3^ environmental sciences^4–6^ and material sciences.^7,8^ From detecting heavy metal traces in water,^9–11^ pesticides in soil^12–14^ to bioactive molecules in biological samples,^1,2,15^ precise molecular analytics is crucial for ensuring (public) safety, production efficiency and research.

Optical spectroscopy is a non-invasive, fast and highly specific technique for molecular analytics. Especially, vibrational spectroscopy is a powerful tool for molecular characterization as it provides a molecular fingerprint without extensive sample preparation. One of the most used vibrational techniques is Raman spectroscopy. Surface-enhanced Raman spectroscopy (SERS) utilizes plasmonic (nano-) structures in vicinity to the analyte molecules enabling even single-molecule detection.^16–18^ The electric field enhancement is strongly linked to the structure of the plasmonic substrate (most commonly made from nanostructured noble metals) which explains the plethora of yearly publications of novel SERS substrates.^17^ Furthermore, this strong structure-enhancement relation is associated with a strong anisotropy in enhancement factors even on the same sample which makes SERS intensities highly unreproducible.^19–21^

Due to the strong localization of the plasmonic near field in gaps or on protrusions, molecules need to be in close proximity to the surface of the metallic nanostructures. In most cases, this is achieved *via* adsorption of the molecule to the (metal) surface. Therefore, most SERS substrates are single-use and prone to contamination leading to high costs and waste generation.^22^ The development of recyclable SERS substrates addresses this issue by enabling multiple uses while ideally maintaining signal intensity.

There are multiple review articles that go into detail of different recycling strategies.^22–25^ In the following section, we will discuss the main principles with selected examples. The simplest idea for substrate recycling is solvent washing. Functionalized Au nanoparticles (NPs) can catch heavy metal ions or volatile organic compounds. Subsequent washing with solvents restores the SERS substrates enabling further use.^23^ Gill *et al.*^26^ report AgNPs on Si nanospikes and test recyclability with the common SERS probe molecule Rhodamine 6G (R6G). Simple washing with water suffices to clean R6G off which enables use for up to 10 recycling steps. A second approach is thermal annealing. High temperature treatments of Au^27–29^ or Ag^30^ substrates can lead to complete removal of analyte molecules. In the latter case, the authors compare solvent washing with thermal annealing and conclude that thermal annealing is crucial for complete R6G removal. However, ethanol washing already removes a major part of the analyte molecules and thermal treatments often restructure the metal surface and heavily change the AEFs. A third recycling method is catalytic cleaning of SERS substrates. These substrates are constructed such that they contain semiconductors which can produce cleaning agents.^22,31^*E.g.* TiO_2_ can be used to make H_2_O_2_ upon UV irradiation to oxidize residue analyte molecules.^32^ However, this requires specially designed SERS substrates which results in a high substrate production effort. For classical Ag and Au SERS substrates, another possibility is recycling *via* chemical reactions. Such SERS substrates can be *e.g.* cleaned upon reactions with NaBH_4_ or H_2_O_2_.^22,33^ Mettela *et al.*^34^ present a similar recycling scheme to that we use in this study: they first convert their Ag to AgCl in HCl and then use ammonia to dissolve and wash off the AgCl from the substrate. This yields again a clean Ag surface which can then be used for further sensing. They report only a slight decrease in Raman intensity after 10 recycling steps. However, this methodology constantly removes a few layers of the silver substrate so that this recycling method slowly decomposes the substrate and produces chemical waste. Subsequent reduction instead of removal of the oxidized layers would be more feasible as it enables indefinite recycling steps in theory. Kang *et al.*^35^ used electrochemical oxidation and subsequent reduction to present a Au substrate with which 40 recycling steps of crystal violet lactone (CV) detection was achieved.

In order to recycle strongly adsorbing analytes, harsher recycling conditions that do not decompose the SERS substrates are required. In this study, we present a plasma-cleaning recycling procedure with which we recycle a simple Ag SERS substrate from strongly adsorbing analytes. Shvalya *et al.*^36^ successfully removed R6G from Au/Pd decorated CuO/Cu_2_O heterostructures with a combined O_2_- and Ar-plasma method. However, they compare their plasma recycling strategy with simple ethanol washing and conclude that simple ethanol washing suffices to remove the majority of R6G. Furthermore, they emphasize that their combined O_2_–Ar-plasma method cannot be applied to Ag based SERS substrates due to fast Ag oxidation. Many further studies were performed where different (oxidizing) plasmas were used to recycle Au SERS substrates.^37–39^ Chevalier and Dwyer^40^ tried O_2_-plasma cleaning of commercially available Au-coated Ag-nanorods. They observed structural changes and studied losses in Raman intensity in different plasma settings. Finally, Okeil and Schneider^41^ present a combined H_2_-/O_2_-plasma treatment of Ag substrates consisting of an initial oxidation in the O_2_-plasma (15 min) with subsequent H_2_-plasma reduction (20 min). They study the change in morphology after this plasma treatment and show SERS spectra of Rhodamine B (RhB). They also mention that this methodology may be used to recycle SERS substrates, but they did not investigate this further.

In the present study, we apply the recycling approach of Okeil and Schneider to a simple Ag SERS substrate which was obtained by thermally evaporating 7 nm of Ag onto a glass cover slip. As probe analytes, we use rhodamine 6G (R6G), 2-mercaptobenzothiazole (MBT) and 1,2-benzene-dithiol (BDT) since they differ in Ag binding strengths. R6G shows only a weak to moderate binding strength towards Ag as it physisorbs *via* its xanthene moiety.^42^ MBT and BDT both chemisorb *via* two heteroatoms forming self-assembled monolayers resulting in stronger adsorptions. MBT is commercially available as anti-corrosion agent emphasizing its metal-binding-strength.^43^ BDT shows the strongest bonding to the Ag substrate through its two neighboring thiol groups, and can generally be considered an extremely strong binder to noble metals. Therefore, we use BDT as benchmark for the presented methodology.

Being able to also recycle substrates covered with BDT increases the area of application for this methodology as the previously discussed studies were only able to recycle substrates covered with weakly or moderately strongly bonding analytes (such as R6G, RhB or CV) where simple solvent washing is already an effective recycling strategy.^26,36^ Our plasma-cleaning based methodology is viable for label-free detection of strongly adsorbing molecules *e.g.* biomolecules or environmental toxins as we are able to recycle a simple Ag substrate for 20 times while consistently being able to record SERS spectra. The obtained Raman enhancement factors are highly reproducible over all recycling steps also yielding a methodology for comparable SERS substrates. Furthermore, the recycling process is solvent-free and produces no waste at all.

## Results and discussion

**Fig. 1:**
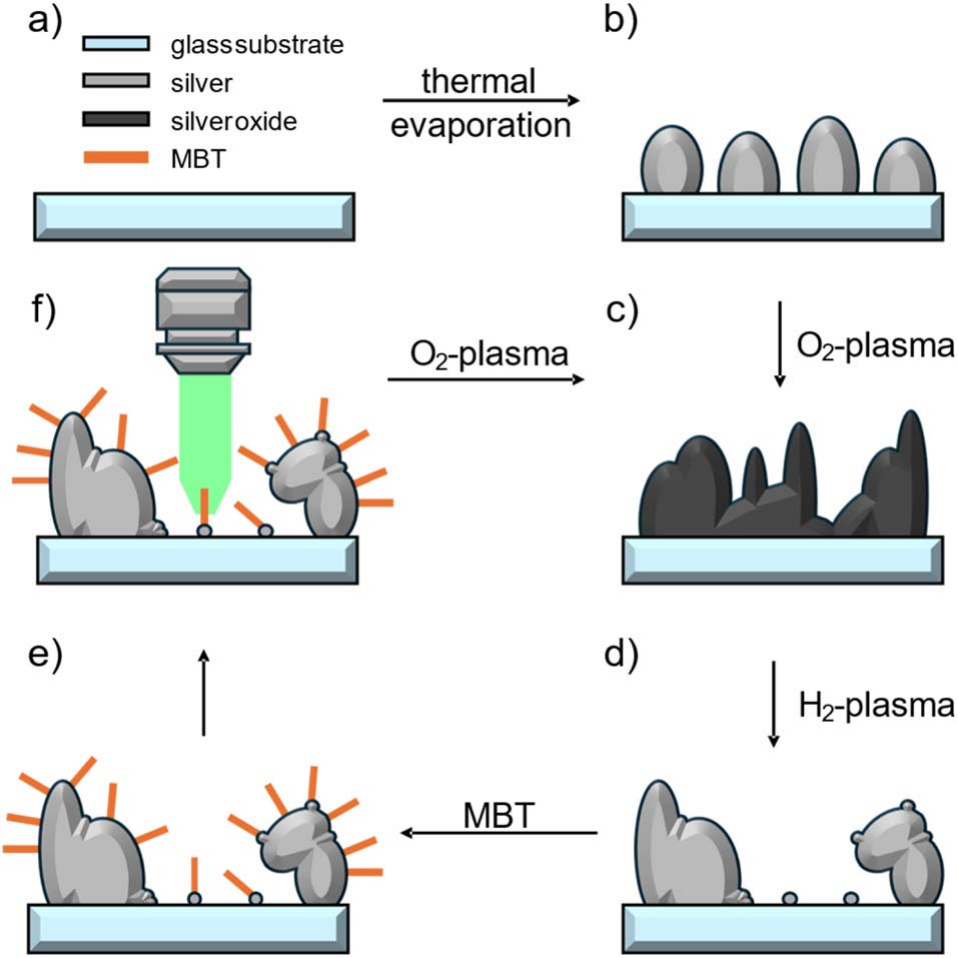
Schematic illustration of the plasma cleaning process. (a) Shows the plasma cleaned glass substrate we use as substrate. Thermal evaporation of Ag leads to (b) a non-closed island film of Ag. O_2_-plasma treatment leads to (c) complete oxidation of the silver removing all organic residues. The resulting silver oxide has a new morphology. H_2_-plasma reduction removes the oxygen yielding (d) a newly structured silver film. (e) Analyte molecules can be deposited which (f) can be detected using SERS. Further plasma cleaning steps can be used to reuse the silver SERS substrates.

The SERS samples are prepared by immersing the Ag substrate in 10^−3^ M analyte solutions with subsequent intense methanol washing. This methanol washing removes higher layers of the analytes ensuring that at least thin layers of R6G and monolayers of MBT and BDT remain. We present a combined O_2_- and H_2_-plasma cleaning treatment for recycling such Ag SERS substrates. Fig. 1 shows a schematic representation of the recycling scheme. The Ag substrate is obtained by thermal evaporation of Ag onto plasma cleaned glass cover slips (Fig. 1a and b). O_2_-plasma treatment leads to complete oxidation of the surface,^44^*i.e.* organic compounds as well as the silver substrate (Fig. 1c). The newly formed silver oxide has a different morphology than the silver islands before. Subsequent H_2_-plasma reduction removes the bound oxygen and yields again a clean Ag surface with a new morphology (Fig. 1d) as we will later show by SEM images. Then, analyte molecules can be attached to the surface and probed *via* SERS (Fig. 1e and f). The plasma recycling scheme can then be further applied to recycle the Ag substrate as the O_2_-plasma completely removes all organic compounds.

One single plasma cleaning step before analyte detection already significantly reduces background signals caused by impurities, *e.g.* amorphous carbon. This makes this methodology not only interesting for multiple substrate uses but can also make a difference in single-use substrates. See chapter S1 and Fig. S1 in the SI for further information.

**Fig. 2:**
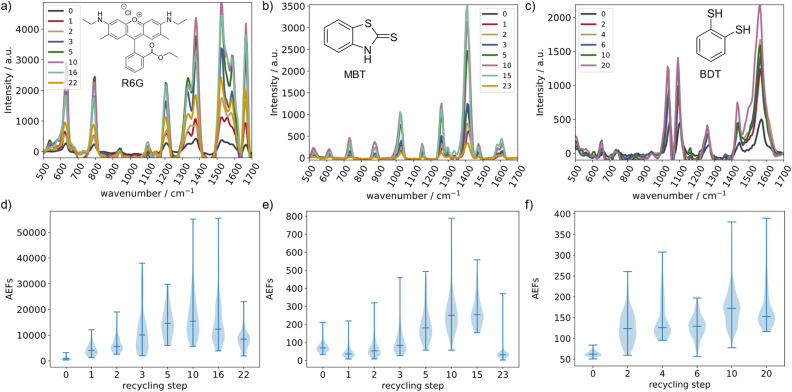
R6G, MBT and BDT SERS spectra of different recycling steps and corresponding AEFs. (a–c) Show the averaged SERS spectra of the different recycling steps. We observe an increase in Raman intensities up to the tenth recycling step and a subsequent decrease. (d–f) Show the corresponding AEFs visualized as violin plots.

We applied this plasma cleaning strategy consisting of first O_2_-plasma oxidation with subsequent H_2_-plasma reduction to recycle our Ag substrates 20 times and remove any R6G, MBT and BDT residues. The corresponding SERS spectra are shown in Fig. 2a–c. BDT shows an extremely strong chemisorption on Ag so that one such plasma recycling step is not enough to restlessly remove it. Therefore, we conducted two subsequent recycling steps in order to completely recycle the Ag substrates. Further details about the recycling effectiveness can be found in chapter S2 and Fig. S2.

In contrast to the recycling studies discussed in the introduction, we report higher Raman signals on substrates that have been recycled ten times. In order to quantify this effect, we estimate analytical Raman enhancement factors (AEFs) for all recorded spectra after each recycling step for a statistically based interpretation. AEFs are estimated as detailed in previous studies.^45,46^1
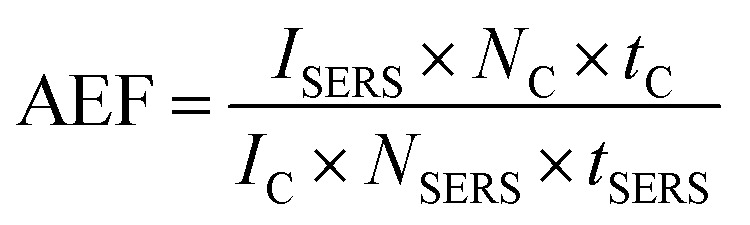


SERS enhancement factors describe the relation between the intensity of SERS peaks and the intensity of the peaks in normal confocal Raman spectra. Therefore, *I*_SERS_/*I*_C_ is the ratio of the SERS and confocal Raman intensities, *N*_SERS_/*N*_C_ is the ratio of number of molecules that contribute to the enhanced Raman spectrum and to the conventional Raman spectrum, and finally *t*_SERS_/*t*_C_ is the ratio of the respective integration times. At this point, we make two assumptions to estimate these AEFs: first, since we do not see any confocal Raman bands even after 5 minutes integration time, we use the noise level as an upper limit for the confocal Raman intensity. Secondly, we assume that the number of molecules in the SERS experiment is equal to the number of molecules in a classical confocal Raman experiment. Since we conduct SERS experiments on SAMs (or at least thin layers), this approximation is again an upper limit to the real conditions. Due to these approximations, the calculated AEFs are a conservative approximation as the real confocal Raman intensities are likely to be significantly lower and the ratio between participating molecules for a confocal and a SERS experiment much higher. For recyclability, however, the exact AEFs are not crucial but their trend as the recycling process progresses.

All three molecules tend to pyrolyze under the conducted experimental settings so that amorphous carbon could almost always be detected. Therefore, we chose peaks with a high Raman intensity outside of the amorphous *C* range (1350–1600 cm^−1^) that can be preferably correlated to one single molecular vibration for AEF analysis. For MBT and BDT, we chose the peaks at 1023 cm^−1^ and 1031 cm^−1^, respectively. Both vibrations can be described as an in-plane deformation of the benzene moiety. For R6G, we chose the carbonyl stretch vibration at 1666 cm^−1^. A more detailed discussion of the chosen peaks by means of DFT calculations can be found in S3 with the calculated spectra shown in Fig. S3.

The AEFs obtained by this method are illustrated in Fig. 2d–f as violin plots. The top and bottom bars show the highest and lowest AEFs while the middle bar represents the distributions' medians. The corresponding box plots as well as an overview table (Table S1) can be found in chapter S4. We observe an increase in AEFs for the first ten recycling steps for all three molecules. The AEFs then decrease again going to the 20th recycling step. For R6G and BDT, we observe a strong increase in AEFs for the first recycling step while MBT shows a decrease. This is due to the fact that the substrate structures are unique to each thermal evaporation process and not controllable. The R6G and BDT substrates were produced in the same batch while that of MBT was produced prior to that. R6G then shows an increase in AEFs up to the tenth recycling step with the strongest increase in AEFs being between the second and the fifth recycling step. The AEF median decreases for the 16th step below the level of the fifth step and then further decreases for the 22nd recycling step. However, the AEF median of the 22nd step is still between that after the second and third step which is significantly increased in comparison to the untreated substrate. MBT follows a similar trend: We first see a strong increase in AEFs for the first ten recycling steps with the strongest increase also being between the second and the fifth recycling step. Further recycling up to 15 recycling steps does not lead to a significant change in AEFs, however, the AEFs drop sharply going to the 23rd recycling step. In this case, the AEFs drop below those of the untreated substrate. BDT also shows the increase in AEFs for the first ten recycling steps, but we do not observe a drastic increase between the second and fifth recycling steps. Furthermore, the AEFs only slightly decrease going to the 20th recycling step, retaining a median over that of the sixth recycling step.

Additionally, we calculate the interquartile ranges (IQRs) of the corresponding box plots as a measure of the distributions' scattering (see Table S1 in chapter S4). The IQR contains all AEFs between 25% and 75% of the distribution. Therefore, the larger the IQR, the larger the AEF scattering. The IQR trend follows the AEF trend: they increase for the first ten recycling steps and decrease again for the 20th recycling step. Both, the three molecules' AEF medians as well as the IQRs correlate with a surface restructuring during the plasma cleaning process. During the first ten recycling steps, the increase in AEFs shows that the restructuring process leads to a surface morphology optimal for SERS enhancement. The increase in IQRs further shows that the inhomogeneity of the surface also increases. Going to the 20th recycling step, both the AEFs as well as the IQRs decrease again hinting towards the degradation of this optimal surface yielding again a more homogeneous surface.

However, the IQRs are in the same order of magnitude for all analytes and all recycling steps so that the AEFs in the IQR can be described as median ± 100%. A deviation of ±100% is common for simple SERS substrates and allows for easy and reliable qualitative sensing. It does not allow for sensing the precise analyte concentration, however, it does allow for differentiating between different orders of magnitude, *e.g.* parts per million and parts per billion.

**Fig. 3:**
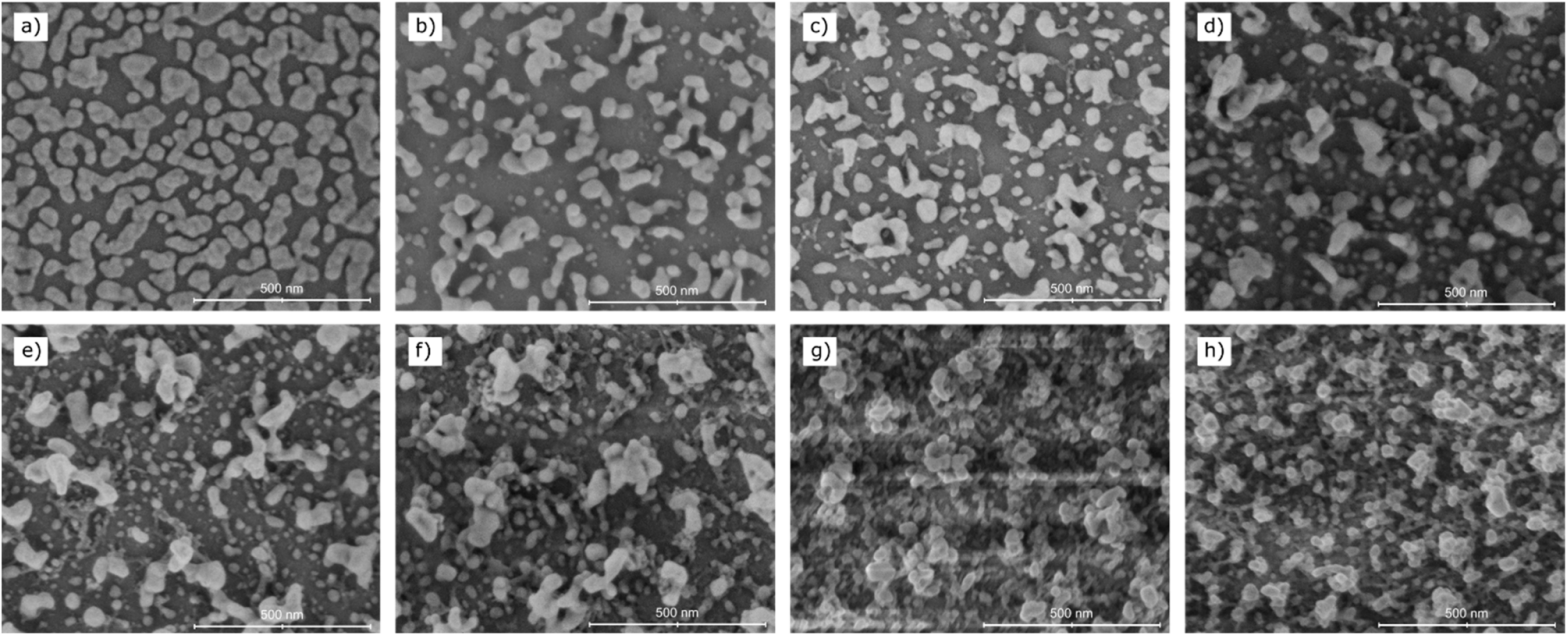
SEM images of the substrate (a) as prepared, and then after (b) 1, (c) 2, (d) 3, (e) 5, (f) 10, (g) 15 and (h) 20 recycling steps. Progressive recycling leads to a restructuring of the silver morphology as the nano island size distribution increases. Starting with the third recycling step, we observe large nano islands that can act as antennas redistributing the excitation energy onto the smaller structures. They can then create strong locally confined electric fields resulting in large AEFs. Furthermore, a web-like structure interconnecting the larger nano islands becomes clearly distinguishable after the fifth recycling step. The SERS spectra show that the surface structure after ten recycling steps is optimal for Raman enhancement. Subsequent recycling steps then start decomposing the larger nano islands back to smaller clusters which is correlated to the decrease in both the AEFs and the IQRs.

For a more detailed understanding of the previously described surface processes, we recorded SEM images for all recycling steps. They are shown in Fig. 3. We observe a pronounced restructuring of the Ag islands between subsequent plasma cleaning steps which is due to the stepwise oxidation and subsequent reduction during the recycling procedure. The O_2_-plasma leads to complete oxidation of the silver layer resulting in a silver oxide layer with a different morphology than the previous Ag layer. The following H_2_-plasma induced oxygen removal yields a fundamentally new morphology of the Ag film.

Comparing the surface of the as-prepared substrate (Fig. 3a) and the surface after the first recycling step (Fig. 3b), we observe less coverage of the glass substrate but more complex and heterogeneous nanostructures. Furthermore, we observe a less isotropic nanoparticle size distribution with particle sizes ranging between a few and several hundred nanometers. Subsequent recycling steps further broaden the nano island size distribution yielding two sets of nano particles: one set of large nano islands that can act as antennas efficiently converting the incident light into plasmonic oscillations and a second set of smaller nano structures with sharp edges and small gaps. The smaller interconnected islands can then be efficiently excited by the larger structures generating high, strongly localized, electric fields between individual nano particles. Such so called hot spots are mainly responsible for high Raman enhancements.^47^ We observe the strongest change in surface morphology between the second and fifth recycling step (Fig. 3c and e) in terms of number and shape of the nano structures. While there is still a continuum of nano structure sizes in the second recycling step, it appears that the two subsets of large nano islands and small nanoparticles separate in the fifth recycling step yielding two non-overlapping sets. This strong change in morphology coincides with the strong change in AEFs for R6G and MBT. Furthermore, the small nano particles also start to form a combined web-like structure interconnecting the large nanometer sized islands in the fifth recycling step. Further recycling steps up to the tenth step do not significantly change the observed surface morphology. Combining the SEM images with the SERS spectra allows us to designate the structure shown in Fig. 3f as the optimal structure of this substrate for Raman enhancement. Further recycling breaks the larger nanoparticles down again into smaller nano islands which coincides with the previously decrease in AEFs. The large nano antennas which are crucial for efficient Raman enhancement are completely decomposed after 20 subsequent recycling steps (Fig. 3h) yielding again one continuous set of nano particles similar to that after three recycling steps. Additionally, we observe a larger surface coverage of intertwined nano structures which dampens hot spot formation.

Yuan *et al.*^48^ studied different Ag surfaces obtained by different plasma oxidation-reduction treatments and calculated the electric field enhancements of the surfaces by both the electrodynamic model as well as the FDTD (finite-difference time domain) method. They modeled individual nanostructures as cylinders capped with a semisphere and found the maximum field enhancement for structures with a cylinder radius of 20 nm and a cylinder length of 60 nm. While we cannot estimate the length of our nano islands from top SEM images, we can estimate the structures' diameter. The second subset of smaller nanoparticles after the fifth recycling step mainly consists of nanoparticles with a diameter of around 50 nm. This is also the case for the sample after the tenth recycling step while further recycling leads to further degradation. After the twentieth recycling step, it appears that most nanoparticles still show diameters in the optimal range, however, there is a much higher coverage of the substrate which might be attributable to a decrease in the structure heights which also negatively impacts the field enhancement.

In the end, we want to highlight that for all recycling steps, the maximum and minimum AEFs differ in only one order of magnitude at the maximum. This is a surprisingly high reproducibility for such simple substrates.

We further expanded our recycling scheme to thermally evaporated Au substrates, however, this recycling scheme is not applicable to analogous Au samples. We discuss this in more detail in chapter S5 by means of SERS spectra (see Fig. S5) and SEM images (see Fig. S6 and S7).

## Conclusions

In this work, we adapt a recycling scheme introduced by Okeil and Schneider^41^ which consists of Ag substrate oxidation in O_2_-plasma with subsequent H_2_-plasma reduction. The studied substrate consists of 7 nm of evaporated Ag on a cleaned glass cover slip. We discuss the surface restructuring processes caused by these harsh conditions by means of SEM images and present SERS spectra of rhodamine 6G, 2-mercaptobenzothiazole and 1,2-benzenedithiol. We show that the recycling procedure is capable of cleaning the substrate without any analyte residue even though MBT and BDT show strong chemisorption on Ag. One initial recycling step already leads to a significant increase in spectrum quality yielding background-free SERS spectra. Further recycling improves the enhancement of the spectra due to further structural changes. The nanostructure size distribution increases for the first three recycling steps until it splits into two subsets of large nano island antennas and small nano particles. Subsequent recycling then decomposes again the nano island antennas yielding one continuous set of nano particles. These structural observations correlate with the reported AEFs. We observe a strong increase in AEFs for the first ten recycling steps which then slowly decrease going the 23rd recycling step. The measured AEFs in all recycling steps only differ in the margin of one order of magnitude which can be viewed highly reproducible. Due to the simplicity of the recycling method, it is widely applicable to all kinds of Ag-based SERS substrates which is a good step towards waste reduction and reproducibility. BDT is likely one of the strongest chemisorbing molecules there is, so with the successful recycling of BDT substrates we can also assume that this method is suited for all conceivable molecules. This is interesting as many environmental toxins, biomolecules and drugs are expected to show strong binding to Ag.

## Experimental methods

### Chemicals

The Ag SERS substrates are obtained by thermal evaporation (Pfeiffer PLS570, 10^−6^ mbar, 0.1 nm s^−1^) of a 7 nm thick Ag layer on top of a plasma-cleaned (Diener Femto, 30 min, O_2_-plasma, *p* = 0.3 mbar) glass cover slip. Rhodamine 6G (R6G) and 2-mercaptobenzothiazole (MBT) were purchased from Sigma-Aldrich and 1,2-benzene-dithiol was purchased from Thermo Fisher Scientific. All chemicals were used without further purification.

For thin layers of R6G on Ag, the substrates are first placed in a 10^−3^ M aqueous solution for 10 minutes and then intensively washed with methanol. Self-assembled monolayers (SAMs) of MBT and BDT are achieved by submersing the Ag substrates for 10 min in a 10^−3^ M methanolic solution. Subsequent intensive washing with methanol ensures the formation of monolayers.

### Recycling method

The Ag SERS substrates were treated in a plasma generator (Diener electronic, Femto) where O_2_ and H_2_ plasmas were created with a firing frequency of 40 kHz and a power of 70 W. The plasma chamber as well as the home-built glass sample mount were always first treated for 30 min with a O_2_-plasma before samples were installed. After installation, we evacuated the chamber to a pressure of 0.16 mbar, then switched on the plasma gas and set the chamber pressure to 0.3 mbar. We ignited the plasma with a power of 70 W and treated the Ag samples for 15 min in O_2_ and for 20 min in H_2_. The chamber temperature was set to room temperature, however, the temperature typically increased to around 50 °C during the plasma treatment.

### SERS measurements

A home-built inverted confocal microscope equipped with a Zeiss α Plan-APOCHROMAT 1.46 NA oil objective, a 532 nm continuous-wave excitation laser (coherent compass 215M) and a Princeton Instruments Acton SP2500 spectrometer (grating density 150 g mm^−1^) followed by a ProEM 512 EMCCD detector were used to obtain SERS spectra. We used different laser powers and integration times for the different analytes. R6G spectra were collected with a laser power of 0.1 mW and an integration time of 1 s. BDT was also excited with a laser power of 0.1 mW but a longer integration time of 10 s was needed. For MBT, we used a laser power of 33 µW with an integration time of 5 s.

At least 150 (up to 230) spectra were automatically recorded on different sample spots for each analyte and recycling step on the entire sample. These spectra were then averaged for clarity and instrumental and fluorescence background corrected. All data processing steps were conducted with Python.

### SEM images

SEM images were taken with a HITACHI SU8030 with an acceleration voltage of 0.5 kV. Both secondary as well as back scattered electrons were analyzed with the HITACHI's upper detector.

## Author contributions

M. B., A. J. M. and K. B. conceptualized and designed the experiments. M. B. and D. H. prepared the MBT samples, conducted the corresponding experiments and did the data analysis. M. B. expanded the experimental methodology to the R6G and BDT measurements as well as all measurements on Au substrates and did the data analysis. All authors discussed the results and participated in editing the manuscript which M. B. wrote.

## Conflicts of interest

There are no conflicts to declare.

## Supplementary Material

NA-008-D6NA00022C-s001

## Data Availability

All data is included in this article and the supplementary information (SI). Raw data can be obtained from the corresponding authors upon reasonable request. Supplementary information is available. See DOI: https://doi.org/10.1039/d6na00022c.
