# Supplementary material for: Recycling Ag SERS-substrates from strongly chemisorbing molecules
Source: Nanoscale Adv. 2026 Mar 12;8(9):2970–6. doi: 10.1039/d6na00022c (PMC13063307; doi:10.1039/d6na00022c)
Supplement: NA-008-D6NA00022C-s001 [file NA-008-D6NA00022C-s001.pdf]

Supplementary Information (SI) for Nanoscale Advances.
This journal is © The Royal Society of Chemistry 2026

Recycling Ag SERS Substrates from strongly chemisorbing Molecules

Marc Bröckel[1,2], Diana Heberle[1], Alfred J. Meixner[1,2] and Kai Braun[1,2]

[1]Institute of Physical and Theoretical Chemistry, University of Tübingen, Auf der Morgenstelle 18, 72076 Tübingen, Germany

[2]Center for Light-Matter Interaction, Sensors&Analytics LISA+, University of Tübingen, Auf der Morgenstelle 15, 72076 Tübingen, Germany

---

Contents

S1. Effect of a single plasma cleaning step before detection 2

S2. Recycling Effectiveness 2

S3. DFT Calculations on R6G, MBT and BDT 3

S4. Statistical AEF analysis via box plots 4

S5. Applying this recycling methodology to Au substrates 6

S1. Effect of a single plasma cleaning step before detection

Figure S1 compares average MBT spectra (averaged over 150 individual spectra) on as-prepared substrates with those on singly plasma cleaned substrates (all spectra are instrumental background corrected). The spectra were collected with a fixed laser power of 33 μW and 5 s integration time. In Figure S1, we observe a broad peak between 2800 cm^{-1} and 2950 cm^{-1} on the as-prepared substrate which we do not observe on the plasma cleaned substrate. These C-H vibrations can be attributed to carbon impurities. Furthermore, in direct comparison, we observe an underlying feature in the area between 1200 and 1600 cm^{-1} on the as-prepared substrate. The peaks there do not go back to zero intensity but remain on a certain background supporting the earlier findings of residual carbon impurities.

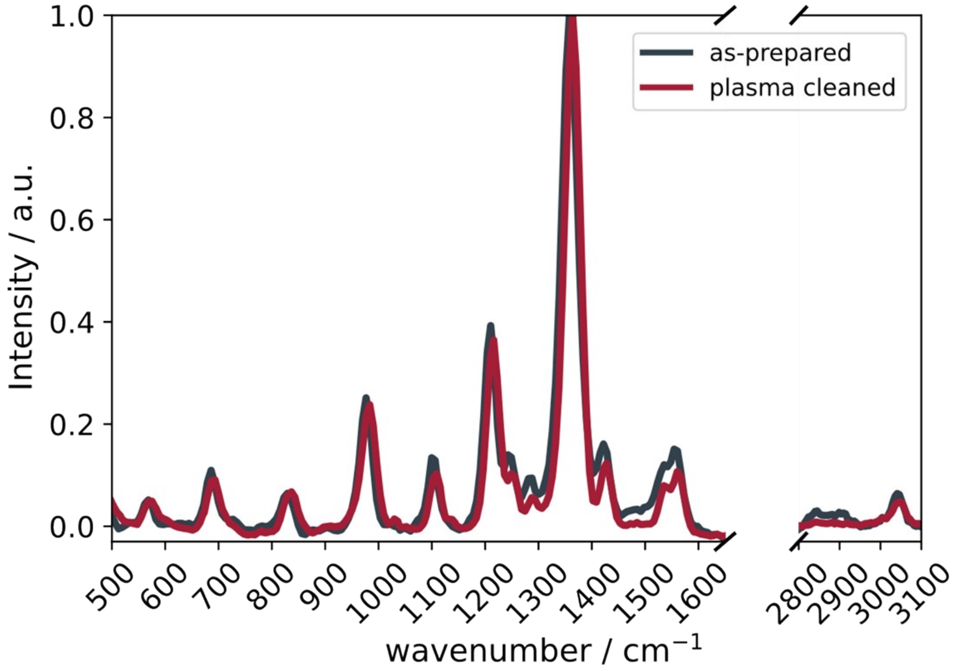

Figure S1: Comparison of the average MBT spectrum (averaged over 150 individual spectra) of the as-prepared Ag SERS substrate by thermal evaporation (black) and the substrate after single plasma cleaning. We observe a broad peak between 2800 and 2900 cm^{-1} as well as an underlying feature between 1200 and 1600 cm^{-1} in the as-prepared sample showing residual carbon impurities. These can be removed by a single plasma cleaning step.
Both spectral features nearly vanish after a single recycling step yielding background free spectra.

S2. Recycling Effectiveness

In order to study recycling effectiveness, we recorded SERS spectra on the freshly cleaned Ag substrates after cleaning from all three analyte molecules. The R6G, MBT and BDT spectra after the first recycling step were collected with a laser power of 80 μW and an integration time of 5 s. To ensure restless cleaning of the BDT substrate, we recorded the SERS spectra after the second recycling step with a laser power of 240 μW. We recorded between 25 and 170 spectra for each substrate at different substrate spots and averaged over them. They were further instrumental background corrected and are shown in Figure S2.

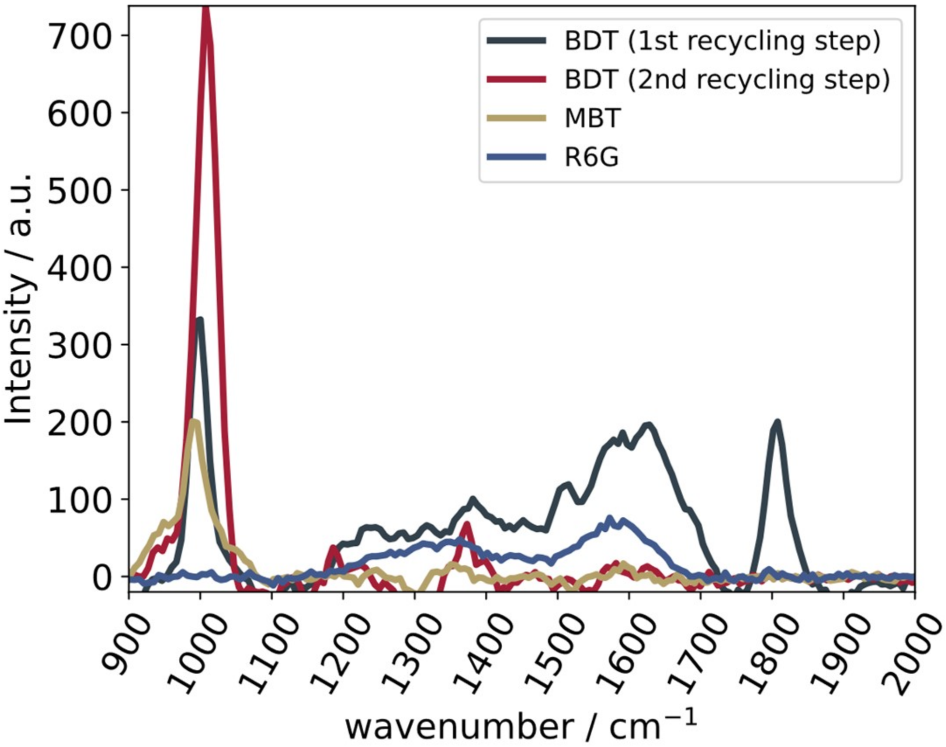

Figure S2: Demonstration of the recycling effectiveness by means of SERS spectra of freshly cleaned Ag substrates. We recorded clean spectra after recycling the substrates from all analyte molecules. For MBT and R6G, one cleaning step suffices to completely remove the analyte. BDT shows significantly stronger chemisorption so that two sequential plasma cleaning steps are required.

For MBT and R6G, we report that one recycling step suffices to completely remove the analyte molecules. The R6G spectrum shows only little amorphous C impurities which are not a residue of the recycling scheme but stem from nascent C by photolysis on the sample. Most R6G spectra show no impurities at all – only long substrate illuminations create these carbon impurities during the measurement. MBT similarly shows only little C contaminations, however, we report a new peak at around 1000 cm^{-1}. To this point, we do not know what this peak stems from. We observe this peak only on bare substrates (not on analyte covered substrates!) but cannot correlate it to literature known Ag or Ag_xO_y peaks.

The spectrum of the BDT substrate after the first recycling step shows a large C signal between 1300 cm^{-1} and 1700 cm^{-1} which partly consists of single resolved Raman bands in the individual SERS spectra. Furthermore, the distinct peak at 1800 cm^{-1} indicates molecular leftovers from the BDT recycling. Therefore, we conclude that a second recycling step is necessary to restlessly remove the BDT from Ag substrates. The substrate after the second recycling step was examined with three times the laser power to ensure that no residue peaks could be detected. Apart from the unidentified 1000 cm^{-1} peak, we see only little amorphous C and no further bands that would indicate BDT leftovers.

S3. <u>DFT Calculations on R6G, MBT and BDT</u>

All calculations were performed using the Gaussian 16 program package.[1] We performed geometry optimizations for all molecules followed by a harmonic frequency calculation to ensure minima structures on the molecules' potential energy surfaces (PESs). R6G and BDT were optimized at the B3LYP[2–5]/def2-TZVP[6], MBT at the B3LYP/6-311++G(d,p)[7,8] level of theory. The harmonic frequencies are scaled with

the anharmonicity factor 0.967[9,10]. Starting from these geometries, (non-resonant) Raman activities are obtainable with the derivative of the polarizability with respect to the atomic displacements in the normal modes. Raman intensities can then be calculated with the excitation laser frequency (532 nm) and the temperature (298.15 K). Even though we are in the resonant Raman regime for R6G, we only computed the non-resonant term as we only want to screen for Raman bands which can be correlated to a single normal mode. Furthermore, we completely neglect the influence of the Ag substrate in these calculations as we are only interested in a band assignment. The obtained Raman intensities are then folded with a

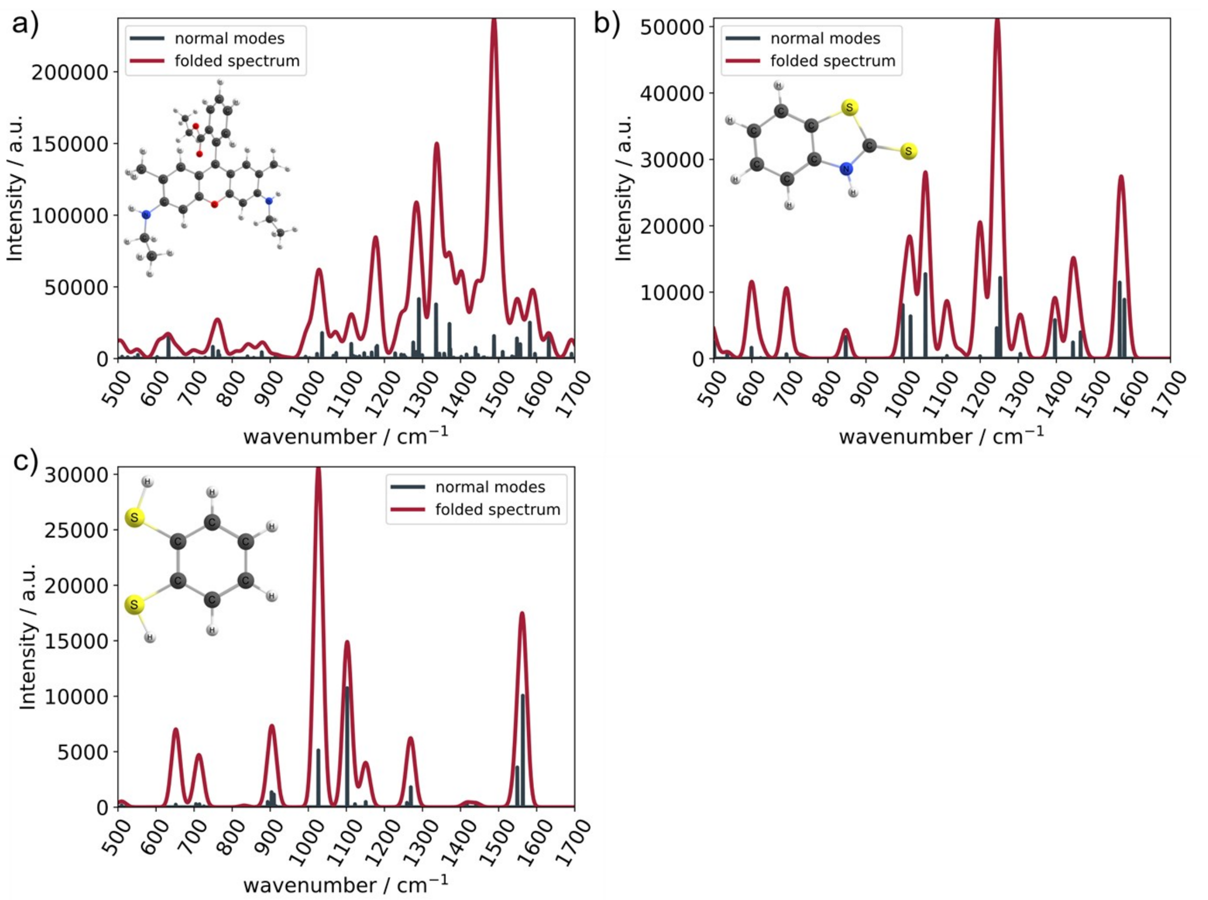

Figure S3: Calculated Raman spectra of a) R6G b) MBT and c) BDT. Black shows the calculated normal modes which were then folded with a gaussian with a FWHM of 20 cm^{-1} to give the red spectrum. The insets show the molecular geometries used for the Raman calculations.

gaussian with a FWHM of 20 cm^{-1}. The obtained Raman spectra are shown in Figure S3. The inlets show the underlying geometries.

These calculations do not correctly describe the real experiment, as we neglect the influence of the Ag substrate as well as the resonant character of the R6G SERS spectrum. However, they suffice to find an experimentally observed band which lies outside the amorphous C range (1350 cm^{-1} – 1600 cm^{-1}) and can ideally be correlated with only one normal mode. For R6G, we choose the band at 1631 cm^{-1} which is the carbonyl C=O stretch. For BDT, we choose the band at 1027 cm^{-1} which can be described as an in-plane deformation of the benzene moiety. MBT shows the corresponding mode at 1014 cm^{-1} which is the band we choose for the AEF analysis. However, in this case, the band cannot be solely described by this one normal mode but a second (in-plane benzene deformation) also lends intensity.

S4. <u>Statistical AEF analysis via box plots</u>

Figure S4 shows the box plots that correspond to the shown and discussed violin plots.

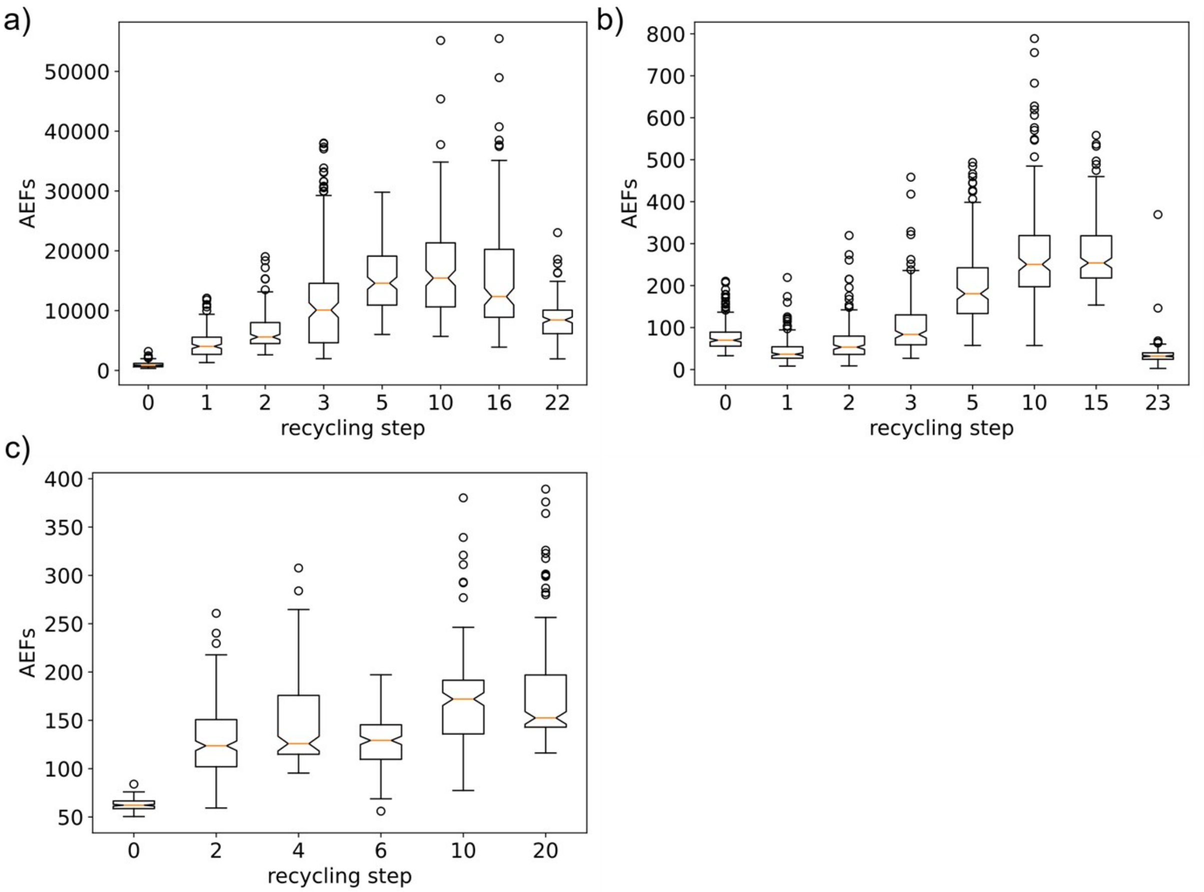

Figure S4: Box plots of the calculated AEFs of a) R6G b) MBT and c) BDT corresponding to the violin plots shown in the main text. The medians are highlighted in orange and the 95 % confidence intervals are illustrated by the boxes' curbs.

The important statistical parameters are shown in Table S1.

Table S1: Statistical parameters of the previously shown box plots.

	R6G			MBT			BDT		
	median	max(AEF)	IQR	median	max(AEF)	IQR	median	max(AEF)	IQR
0	989	3187	575	79	211	33	62	84	8
1	4703	14793	2878	46	219	27			
2	6565	19028	3510	67	319	44	124	261	49
3	11966	38020	9937	104	458	71			
4							126	208	61
5	15177	29796	8192	205	493	109			
6							129	197	36
10	17566	55164	10710	273	789	122	172	380	56
15 / 16	15324	55482	11356	273	558	101			
20 / 22 / 23	8290	23040	3930	35	369	15	153	389	54

The interquartile range (IQR) is the range in which 50% of all values lie. We use it in this study as a measure of the distributions' broadness. The larger the IQR the larger the scattering of the AEFs. The values are discussed in the main text.

S5. Applying this recycling methodology to Au substrates

Similarly to the Ag substrates, we produced Au substrates by thermal evaporation with the same technical properties discussed for the Ag samples. We also applied MBT to the Au substrates following the same sample preparation strategy and employed the same recycling scheme. SERS spectra were recorded with a 633 nm laser (Uniphase 1137P) with a power of 250 µW and an integration time of 5 s. Averaged MBT SERS spectra (where the Au luminescence background was removed) of the untreated

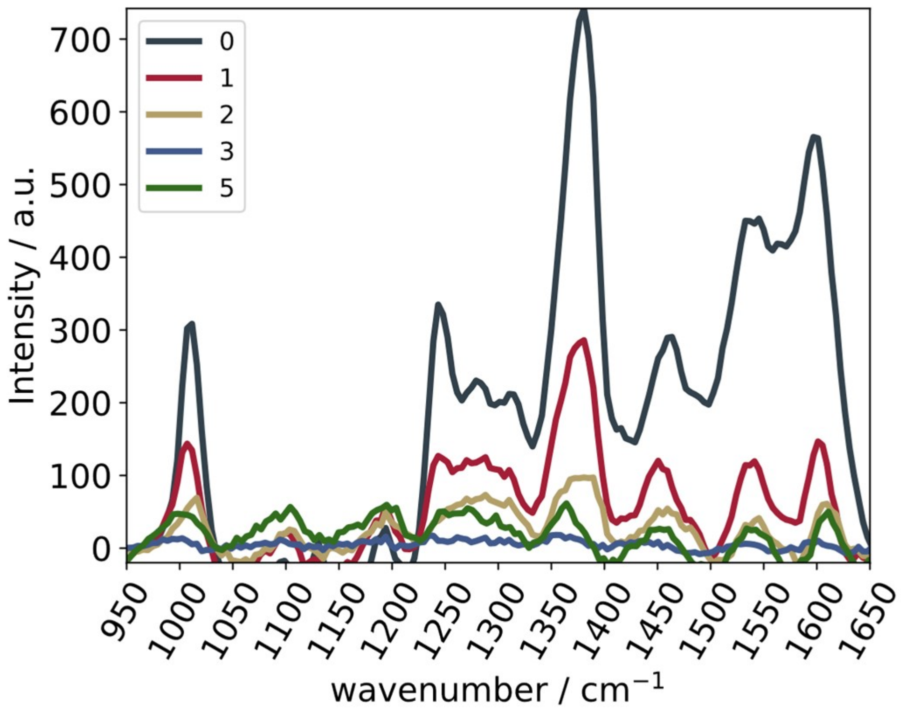

Figure S5: MBT SERS spectra on the as-prepared sample in comparison to the spectra after the different recycling steps. After the second recycling step, we do not see any MBT anymore.

Au substrate as well as the substrates after different plasma cleaning steps are shown in Figure S5.

We report MBT spectra on the untreated Au substrate which show significantly lower intensities than the corresponding Ag substrates due to the lower Raman enhancement. We also observe high Raman intensities of the two peaks between 1500 cm^{-1} and 1650 cm^{-1} which are likely due to amorphous C contaminations. A single recycling step again suffices to restlessly remove the MBT. However, the MBT SERS spectra are barely recognizable on the one-time recycled substrate. And further recycling steps lead to the complete loss of Raman signal. The sinusoidal background pattern observed in the spectra of the second, third and fifth spectrum are not linked to the molecule but stem from an interference at the 633 nm long range filter. This is unexpected as we would have guessed that these analogous Au substrates just behave like their Ag counterparts. We recorded the SEM images shown in Figure S6 to understand the restructuring processes of the substrate.

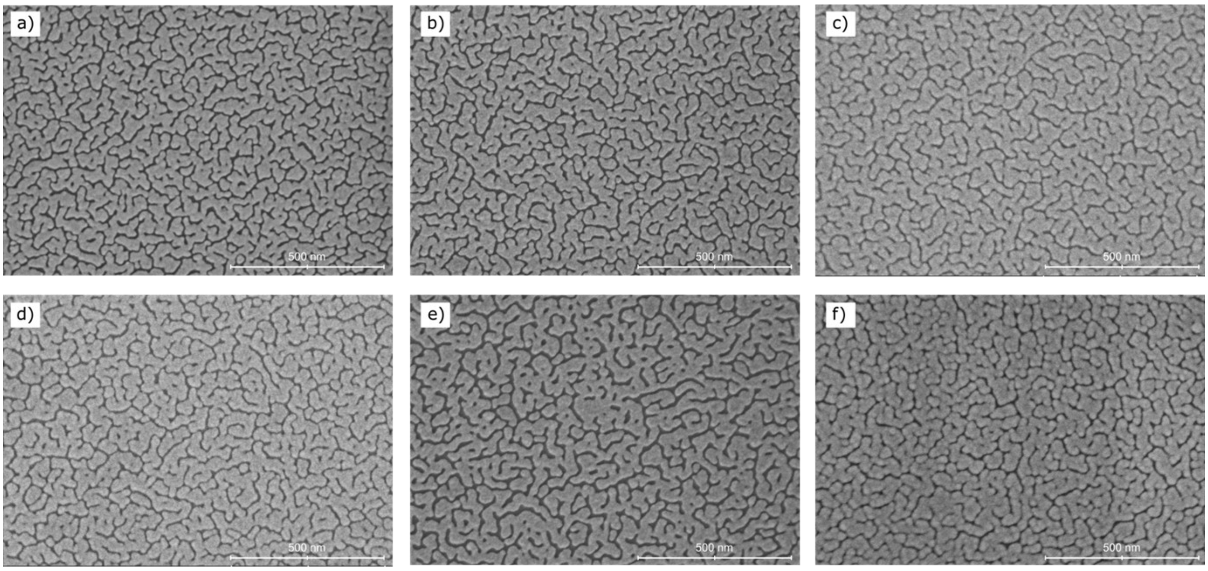

Figure S6: SEM images of the substrate a) as prepared and then after b) 1, c) 2, d) 3, e) 5 and f) 10 recycling steps. In contrast to the Ag sample, we do not observe a strong restructuring of the Au surface during the recycling steps.

In contrast to Ag, Figure S6 shows no significant restructuring of the Au surface during the recycling

steps. We see a widely connected network of Au nano islands with consistent interstructural distances. Even though the SEM does not provide height information, the contrast can sometimes be interpreted as such. Therefore, we cautiously like to point out that with subsequent recycling steps, we observe brighter spots at the edges of the nano islands and think that these might be protrusions that stand out of the islands. Without any further confirmation from AFM or STM measurements, we think that the nano islands get rougher during the recycling scheme. This should in principle lead to an increase in Raman intensities and these SEM images do not provide yet an explanation as to why the Raman signal vanishes after the second recycling step. During the SEM experiments, we further investigated the edges of scratches on the Au substrates and found the following behavior on the sample that was five-times recycled.

Figure S7 a) shows the SEM images of such a scratch and interestingly we observe sharp edges of a closed film underneath the Au nano islands. Without any further experimental evidence, we reckon that this film must be a closed Au film as we did not use any adhesion layer or different metal during substrate preparation. Figure S7 b) shows a zoom into the most prominent edge. Then, we went back to our SEM samples and cautiously scratched them with a thin needle. Figure S7 c) – e) show the corresponding SEM images of the c) as-prepared substrate d) the substrate after one recycling step and e) the substrate after two recycling steps. We do not observe any film on the untreated sample. The dark grey area to the right of the nano islands is pure glass which we use as substrate basis. After one recycling step, we observe no sharp edges on the substrate as in the case of Figure S7 a), but we do not observe a flat surface next to the nano islands anymore. We observe small protrusions on the surface where the nano islands priorly were and it appears that they are on top of a closed film. The same effect can be seen on the two-times recycled substrate. We did not investigate this effect any further by means of XPS or EDX in the scope of this work, but we would cautiously interpret these results as a closed Au film formation underneath the nano islands due to the plasma treatment. This would explain the

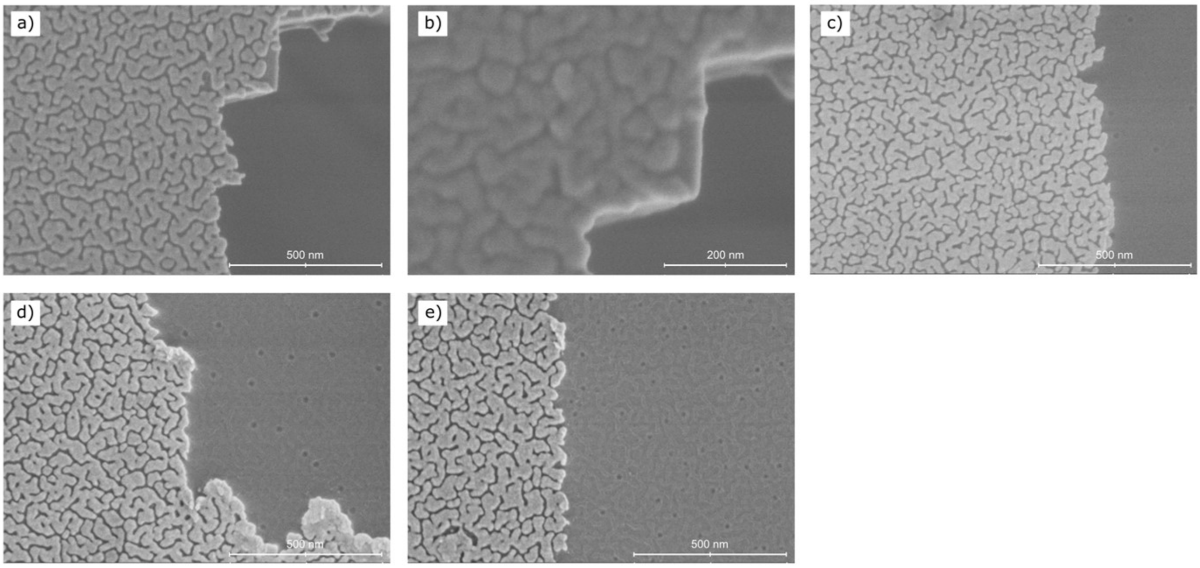

Figure S7: SEM images of scratches on different Au samples. a) was recorded on the five-times recycled substrate. We see a closed film underneath the nano islands. b) shows a zoom into the upper right corner of the underlying film. c) was then recorded on the untreated substrate where we observe no such film. d) and e) were recorded on the one-time and two-times recycled substrates and we slowly see an emerging closed film.

decreasing SERS AEFs in the experiment as the closed Au layer would quench any local electric fields resulting in little to no Raman enhancement. Furthermore, this observation would correlate with the observation that the as-prepared substrate is not electrically conductive, but starting with the one-time recycled substrate, we measure a small electric conductivity (~ kΩ).

(1) Frisch, M. J., Trucks, G. W., Schlegel, H. B., Scuseria, G. E., Robb, M. A., Cheeseman, J. R., Scalmani, G., Barone, V., Petersson, G. A., Nakatsuji, H., Li, X., Caricato, M., Marenich, A. V., Bloino, J., Janesko, B. G., Gomperts, R., Mennucci, B., Hratchian, H. P., Ortiz, J. V., Izmaylov, A. F., Sonnenberg, J. L., Williams-Young, D., Ding, F., Lipparini, F., Egidi, F., Goings, J., Peng, B., Petrone, A., Henderson, T., Ranasinghe, D., Zakrzewski, V. G., Gao, J., Rega, N., Zheng, G., Liang, W., Hada, M., Ehara, M., Toyota, K., Fukuda, R., Hasegawa, J., Ishida, M., Nakajima, T., Honda, Y., Kitao, O., Nakai, H., Vreven, T., Throssell, K., Montgomery, J. A., Jr., Peralta, J. E., Ogliaro, F., Bearpark, M. J., Heyd, J. J., Brothers, E. N., Kudin, K. N., Staroverov, V. N., Keith, T. A., Kobayashi, R., Normand, J., Raghavachari, K., Rendell, A. P., Burant, J. C., Iyengar, S. S., Tomasi, J., Cossi, M., Millam, J. M., Klene, M., Adamo, C., Cammi, R., Ochterski, J. W., Martin, R. L., Morokuma, K., Farkas, O., Foresman, J. B., Fox, D. J. Gaussian 16.

(2) Vosko, S. H.; Wilk, L.; Nusair, M. Accurate Spin-Dependent Electron Liquid Correlation Energies for Local Spin Density Calculations: A Critical Analysis. *Can. J. Phys.* **1980**, *58* (8), 1200–1211. https://doi.org/10.1139/p80-159.

(3) Stephens, P. J.; Devlin, F. J.; Chabalowski, C. F.; Frisch, M. J. Ab Initio Calculation of Vibrational Absorption and Circular Dichroism Spectra Using Density Functional Force Fields. *J. Phys. Chem.* **1994**, *98* (45), 11623–11627. https://doi.org/10.1021/j100096a001.

(4) Lee, C.; Yang, W.; Parr, R. G. Development of the Colle-Salvetti Correlation-Energy Formula into a Functional of the Electron Density. *Phys. Rev. B* **1988**, *37* (2), 785–789. https://doi.org/10.1103/PhysRevB.37.785.

(5) Becke, A. D. Density-functional Thermochemistry. III. The Role of Exact Exchange. *J. Chem. Phys.* **1993**, *98* (7), 5648–5652. https://doi.org/10.1063/1.464913.

(6) Weigend, F.; Ahlrichs, R. Balanced Basis Sets of Split Valence, Triple Zeta Valence and Quadruple Zeta Valence Quality for H to Rn: Design and Assessment of Accuracy. *Phys. Chem. Chem. Phys.* **2005**, *7* (18), 3297–3305. https://doi.org/10.1039/B508541A.

(7) McLean, A. D.; Chandler, G. S. Contracted Gaussian Basis Sets for Molecular Calculations. I. Second Row Atoms, Z=11–18. *J. Chem. Phys.* **1980**, *72* (10), 5639–5648. https://doi.org/10.1063/1.438980.

(8) Krishnan, R.; Binkley, J. S.; Seeger, R.; Pople, J. A. Self-Consistent Molecular Orbital Methods. XX. A Basis Set for Correlated Wave Functions. *J. Chem. Phys.* **1980**, *72* (1), 650–654. https://doi.org/10.1063/1.438955.

(9) Johnson III, R. D. NIST Computational Chemistry Comparison and Benchmark Database, NIST Standard Reference Database Number 101, 2022. http://cccbdb.nist.gov/.

(10) Kesharwani, M. K.; Brauer, B.; Martin, J. M. L. Frequency and Zero-Point Vibrational Energy Scale Factors for Double-Hybrid Density Functionals (and Other Selected Methods): Can Anharmonic Force Fields Be Avoided? *J. Phys. Chem. A* **2015**, *119* (9), 1701–1714. https://doi.org/10.1021/jp508422u.
